# Mobile device location data reveal human mobility response to state-level stay-at-home orders during the COVID-19 pandemic in the USA

**DOI:** 10.1098/rsif.2020.0344

**Published:** 2020-12-16

**Authors:** Chenfeng Xiong, Songhua Hu, Mofeng Yang, Hannah Younes, Weiyu Luo, Sepehr Ghader, Lei Zhang

**Affiliations:** 1 Maryland Transportation Institute (MTI), University of Maryland, College Park, MD 20742, USA; 2 Center for Shock, Trauma, and Anesthesiology Research (STAR), University of Maryland, Baltimore, MD 21201, USA

**Keywords:** human mobility, COVID-19, mobile device location data, behavioural response

## Abstract

One approach to delaying the spread of the novel coronavirus (COVID-19) is to reduce human travel by imposing travel restriction policies. Understanding the actual human mobility response to such policies remains a challenge owing to the lack of an observed and large-scale dataset describing human mobility during the pandemic. This study uses an integrated dataset, consisting of anonymized and privacy-protected location data from over 150 million monthly active samples in the USA, COVID-19 case data and census population information, to uncover mobility changes during COVID-19 and under the stay-at-home state orders in the USA. The study successfully quantifies human mobility responses with three important metrics: daily average number of trips per person; daily average person-miles travelled; and daily percentage of residents staying at home. The data analytics reveal a spontaneous mobility reduction that occurred regardless of government actions and a ‘floor’ phenomenon, where human mobility reached a lower bound and stopped decreasing soon after each state announced the stay-at-home order. A set of longitudinal models is then developed and confirms that the states' stay-at-home policies have only led to about a 5% reduction in average daily human mobility. Lessons learned from the data analytics and longitudinal models offer valuable insights for government actions in preparation for another COVID-19 surge or another virus outbreak in the future.

## 1. Introduction

The 2019 coronavirus disease (COVID-19) pandemic is undoubtedly one of the worst global health crises seen in decades. The first confirmed case of COVID-19 in the USA emerged in Washington State on 21 January 2020. Three months later, over three-quarters of a million cases had been confirmed throughout the nation. Governments around the world are taking rapid action to mitigate the spread of the disease. The US government proclaimed a national state of emergency on 13 March 2020. Following an exponential growth in the number of confirmed cases, the Federal Emergency Management Agency (FEMA) announced its first major disaster in the state of New York on 20 March 2020, followed by California and Washington on 22 March [1]. As of 11 April 2020, FEMA had announced that the COVID-19 pandemic was a disaster in every state, with Wyoming being the last one. On 19 March 2020, California became the first state to institute a stay-at-home or shelter-in-place order [2]. By mid-April 2020, all but eight states had followed suit. Three of the eight states had partial stay-at-home orders, implemented by city mayors or county executives [3].

One approach to delaying the spread is to reduce human travel by imposing travel restriction or lockdown policies, with the aim of possibly decreasing the rate of case exportations [4–7]. Government appropriations are a crucial component in reducing case fatalities in the USA [4,8]. The research that focused on analysing the impact of such policies on the spread of COVID-19 generally uses epidemic models, such as the susceptible–infectious–recovered (SIR) model and its extensions [9–11]. By assuming a universal magnitude of mobility reduction, the impact of different lockdown or travel restriction policies can be quantified. However, people's compliance with such policies is different across regions and cannot be assumed as a single factor [7]. For instance, research suggests that lockdowns or travel restrictions do not have significant effects in developing countries compared with developed countries, and they would work only if the opportunity costs of staying at home are not too high [5]. It is still unclear how effective these policies are in suppressing the mobility trend to flatten the curve, largely because of the lack of an observed and large-scale dataset describing human mobility patterns during the pandemic.

Mobile device location data have become popular for studying human mobility. Earlier studies with mobile device location data mainly used either GPS technology (e.g. [12–14]), call detail records [15,16] or location-based social media data (e.g. [17]) to study individual-level mobility. Mobile device location data have also been used in several COVID-19-related studies, including a few platforms produced by companies where the only travel distance is measured (e.g. [18–20]). Largely because of the lack of data sources and data analytical algorithms, these previous studies suffer from notable limitations in three aspects. First, we miss the comprehensive and granular analysis of individual-level measurements: we cannot tell whether people are staying at home or making more trips. Second, research that highlighted the importance of staying at home and practising social distancing often assumed the levels of compliance in reducing travel. This actually can be empirically verified and/or adjusted through observed data. Third, there might be spontaneous and natural mobility changes due to the panic of COVID-19, in addition to the reaction to government orders. A data-driven and longitudinal model is imperatively needed to capture an individual's reaction to the disease and social distancing orders, especially in advance of future COVID-19 outbreaks where prolonged or intermittent social distancing might be required [21]. Taking advantage of emerging mobile device location data and cloud computing resources, this paper aims to present some steps to overcome this knowledge gap.

## 2. Data

Compared with prior studies, this paper for the first time develops data analytics to derive individual trip-level measurements to quantify and statistically model the human mobility response to COVID-19 and the stay-at-home state orders in the USA. To capture the dynamic behaviour response, a data panel of integrated and processed location data representing movements for the entire USA was developed, incorporating daily movements of about 20 million anonymous individuals from 1 January 2020 to 11 April 2020 (over 150 million monthly active samples). Then, a cloud-based computing platform was deployed using a validated spatial–temporal algorithm [22], ingesting over 60 TB of data and using over 75 000 CPU hours of computation to identify all trips. A multi-level weighting procedure expanded the sample to the entire US population, using device-level and trip-level weights to ensure data representativeness across the nation, states and counties. All computed metrics are made available to the general public via the COVID-19 analysis platform developed by the authors (https://data.covid.umd.edu/).

The data panel and the computational algorithms have been validated based on a variety of independent datasets, such as the National Household Travel Survey and the American Community Survey, and peer-reviewed by an external expert panel in a US Department of Transportation Federal Highway Administration's Exploratory Advanced Research Program project [22]. More details about the data, analytics and descriptive statistics can be found in the electronic supplementary material, Section I. Overall, three human mobility metrics were developed at the national and state level and integrated with COVID-19 case data collected from the Johns Hopkins University COVID-19 dashboard [23], population data and other data sources for the analyses [24,25].

— Daily average number of trips per person: the total number of identified and weighted trips in each day that are beyond 985 feet (300 m) in length, divided by the population.
— Daily average person-miles travelled (PMT): the total weighted PMT in each day across all travel modes, divided by the population.
— Daily percentage of residents staying at home: a person is considered to be staying at home if she/he makes no trip longer than 1 mile (1 mile = 1.6 km) away from home. A trip with both trip-ends at home (e.g. jogging 3 miles) does not violate our staying-at-home criteria.

**Figure 1.**
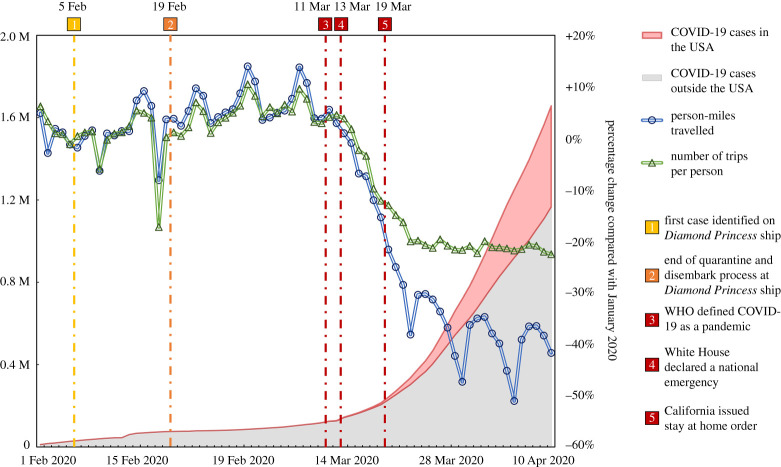
National human mobility trend during the COVID-19 crisis, quantified by daily average number of trips per person and daily average person-miles travelled.

Using the human mobility metrics in January 2020 as the comparison benchmark (New Year's Day, 1 January, and Martin Luther King Jr. Day, 20 January, are excluded), we quantified the national mobility trend during COVID-19, as visualized in figure 1. People did not reduce their travel until the second week of March, during which the World Health Organization (WHO) defined COVID-19 as a global pandemic and the proclamation on declaring a national emergency was made by the White House [26]. Immediately after, the sharpest deterioration of mobility was observed in the third week of March when California issued its stay-at-home order.

Following California and after about two weeks into the pandemic (mid- to late March), over 40 states began issuing their own travel restrictions. However, it is clearly seen in our metrics that these government restrictions failed to reduce human mobility in two ways. First, American people had already spontaneously limited their travel well before the issuance of state directives. For instance, the average daily number of trips per person in the USA had already tumbled 15% before the first state entered stay-at-home status (figure 1). Second, a ‘floor’ on human mobility was observed; even after the issuance of the orders, the mobility decrease had stagnated. In fact, the average daily number of trips per person has never fallen by more than 23% across the nation. We will expand on this topic with more analytics and modelling.

## 3. The failure of the stay-at-home orders in the USA

The above-mentioned two sources of failure can be measured quantitatively via our mobile device location data analytics.

**Figure 2.**
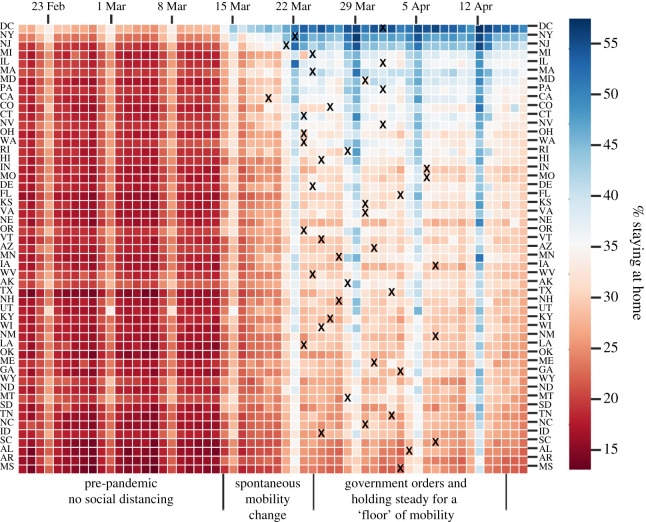
Daily percentage of state residents staying at home by state (20 February–17 April data used from data.covid.umd.edu; ‘X’ indicates a state-wide stay-at-home order issuance).

First, we visualized the daily percentage of state residents staying home by state in figure 2. We had observed *spontaneous mobility change* before the nation entered emergency status and different states issued stay-at-home orders. In most states, this spontaneous reduction was more significant than the data observed after the statewide stay-at-home order issuance, marked with ‘X’ in figure 2. Further, 8 out of 43 states that announced stay-at-home orders had the maximum mobility drop before the order issuance. For instance, the District of Columbia had the highest percentage of people staying at home in the nation. That percentage increased from 27% to 54% within two weeks in mid-March (before the order came out). It is hypothesized that the facts about the virus outbreak, such as the daily number of new infections in the nation, are sufficiently persuasive for people to practise self-quarantine. This is tested in the statistical models presented later in the paper.

Second, by the time stay-at-home orders were issued by state governors, people were already practising social distancing. The changes in human mobility had then stagnated even with a stay-at-home order imposed and held steady to form a lower bound, i.e. *the ‘floor’ on human mobility*. For instance, except for the District of Columbia, New York and New Jersey, the daily percentage of residents staying at home in the majority of states never exceeded 50%. The daily average number of trips per person has never fallen by more than 25% in 19 different states. The highest increase in the percentage of people staying at home during the week after a state-wide order compared with the week before the order belongs to New Jersey (13%), followed by New York (11%), Illinois (11%), California (11%) and Michigan (10%). On average, states with a state-wide stay-at-home order as of 10 April have a 5.6% higher increase in the percentage of people staying at home than the states that did not have a stay-at-home order at that time. Compared with the *spontaneous mobility change* where a 27% higher increase was observed in the percentage of people staying at home within two weeks in mid-March, the issuance of stay-at-home orders only contributed marginally to approach the mobility ‘floor’. This is then verified in the modelling practice presented in the next section.

**Figure 3.**
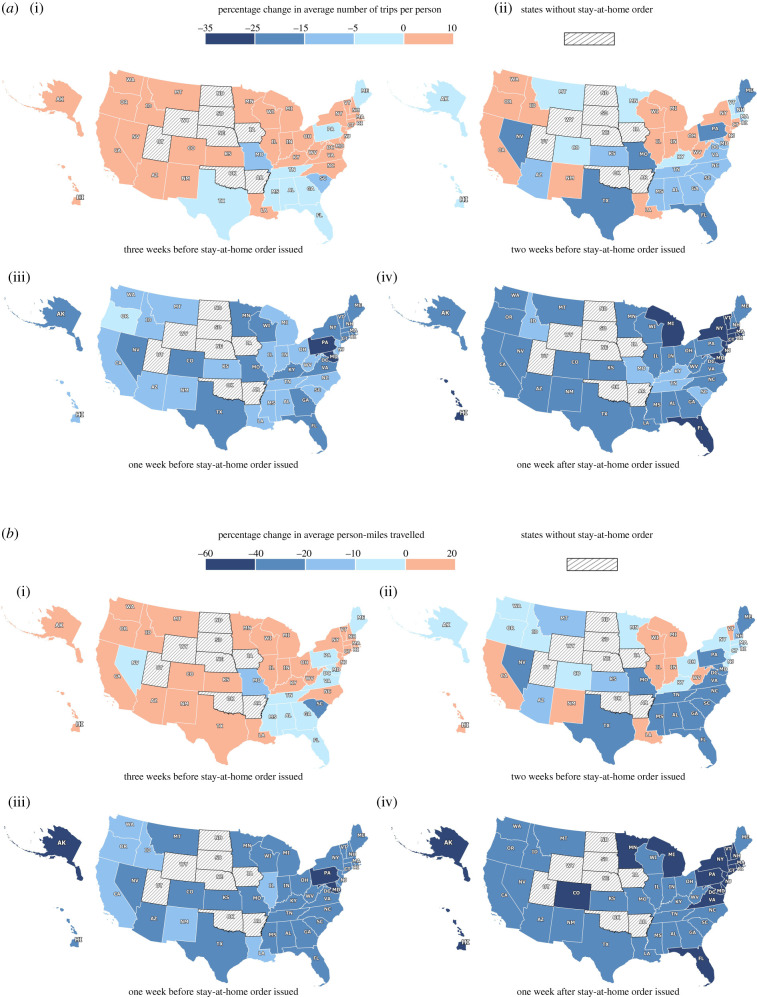
Change in (*a*) average number of trips per person and (*b*) average person-miles travelled one, two and three weeks before and one week after a stay-at-home order took effect relative to January 2020.

By 10 April, only eight states had not issued stay-at-home orders. We reorganized the mobility data and anchored the data to the dates when different states announced their stay-at-home orders and then quantified the human mobility changes compared with the January benchmark: three weeks, two weeks and one week before and after the orders were issued (illustrated in figure 3). Though limited, stay-at-home orders did show positive effects in reducing people's travel and travel distances. Our analytics depict that an additional 6.1% decrease in the daily average number of trips per person and 10.8% decrease in daily average PMT were observed in the week immediately after the order took effect across different states, compared with the week before (as depicted in figure 3*a,b*). The states that saw the most significant mobility drop after the orders are New Jersey (−17% trip, −23% PMT), New York (−17% trip, −22% PMT), Illinois (−16% trip, −23% PMT), California (−16% trip, −22% PMT) and Michigan (−14% trip, −23% PMT). The least significant mobility drops (±5%) can be observed in Pennsylvania, Kentucky, Missouri, Tennessee and the District of Columbia. Miles travelled per person after a state-wide order, in comparison with the week before the order, declined by 9.7 miles in Illinois, followed by California (9 miles), New Jersey (8.9 miles), Hawaii (8.5 miles) and New York (8.5 miles). The lowest declines in miles travelled per person following the orders are observed in Missouri (−1.9 miles, so actually an increase), South Carolina (−0.9 miles), Pennsylvania (0.3 miles), District of Columbia (1.1 miles) and Texas (1.3 miles). On average, the policy has reduced miles travelled per person from 27.1 to 22.6 miles per person.

Stay-at-home orders vary widely in when, where and how they are implemented and enforced. For instance, essential activities, such as receiving healthcare services, going grocery shopping, exercising and essential work, are authorized in the orders. Differences in the orders emerge in religious gatherings, where some states allow for gatherings of more than 10 people for special events but most do not. Interstate travel is sometimes allowed, although some states, such as Alaska, require special permission to leave the state and others, like Nevada, require a 14-day quarantine period for anyone re-entering the state [27,28]. Education institutions have been ordered to close in nearly half the US states for the remainder of the year. Only seven states have recommended that schools be closed as opposed to ordering closure altogether [29]. Seeing family members outside of their immediate household is also not permitted in several states, unless it is for ‘caring for the family member’, i.e. providing groceries for or medical aid to elderly or immunocompromised family members. Additionally, not all stay-at-home orders are implemented at the state level. Many were deployed in counties or cities before the entire state adopted the order. As of mid-April, three states–Oklahoma, Utah and Wyoming—had partial orders.

Enforcement is a crucial component of stay-at-home orders. Fifteen states (including the District of Columbia) have stated that they would issue fines and possible jail time for persons violating the order; 14 have stated that they would enforce the order by first issuing a warning, then possibly issuing a fine for a repeated offense; 14 more either have not stated how the order would be enforced or have explicitly stated that it would only be enforced through education, without penalties [30]. This variation in how the order is enforced conveys a possible discrepancy in how people respond to the order. Risking a fine of $1000 or above and possible jail time in New Jersey, Hawaii, the District of Columbia and several other states may deter residents from going out unnecessarily more so than states that solely focus on educating people who violate the order.

Overall, government stay-at-home orders have not accomplished significant human mobility responses according to our data analysis. More descriptive statistics on how mobility has changed (over time) before and after a state-issued stay-at-home order are available in electronic supplementary material, Appendix, Section I. The spontaneous mobility reduction phenomenon suggests that people who were able to practise social distancing had already done so before government intervention. The ‘floor’ on human mobility indicates that those who could not or were not willing to stay at home has rendered government orders less effective than the spontaneous mobility change. A closer look at these metrics in different states also indicates that individuals from different states exhibit heterogeneity in mobility responses, and the government orders may suppress a varied effect in regulating travels. Time-dependent longitudinal modelling at the state level will help quantify the mobility response and the effectiveness of different state government orders.

## 4. Methods and results

### 4.1. Model description

To quantify how people in different states responded to stay-at-home state orders during the COVID-19 pandemic, we studied the longitudinal changes in state-level mobility using a generalized additive model (GAM) [31,32] of the daily average number of trips per person and daily average PMT. A notable advantage of GAM lies in its flexibility, which combines the linear and fixed effects with nonlinear effects, such as temporal patterns, variable interaction and individual-specific random effects. These effects are realized via the additive terms of GAM, including (i) random effects across all states to capture the unobserved heterogeneity; (ii) interactions between the orders and states to capture the varying effect of policies across different states; (iii) time-varying patterns, including an average changing pattern and a weekly changing pattern, to estimate the autoregressive time series; and (iv) spatio-temporal interactions to capture the spatio-temporal heterogeneity over time and across different states. These are essential in the longitudinal analysis since observations are intra-subject correlated, time-varying and with unobserved heterogeneity. The formulation of our GAM is shown as follows:

4.1 $$\begin{array}{rl} T_{it} & =\beta_{0}+\overset{K}{\underset{k=1}{\sum }}\beta_{ik}X_{ikt}+\overset{L}{\underset{l=1}{\sum }}f_{il}(X_{ilt})+\overset{R}{\underset{r=1}{\sum }}\overset{S}{\underset{s=1}{\sum }}{\;\tilde{f}}_{ir}(X_{irt})\\ & \;\times {\;\tilde{f}}_{is}(X_{ist})+\;f_{i}^{\prime }(b_{i})+\vartheta_{it}, \end{array}$$

where

— *T_it_* is the average number of trips per person or average PMT in state *i* on day *t*;
— *K* is the total number of fixed effects; $X_{ikt}$ refers to the *k*th covariate in county *i* on day *t*; these covariates include key independent variables used in the model, such as the daily number of newly confirmed coronavirus cases and the level of enforcement of the state orders;
— *β*_0_ is the overall intercept; $\beta_{ik}$ is the *k*th coefficient of fixed effects that vary across different states.
— $f_{il}(.)$ is a low-rank isotropic smooth function and $X_{ilt}$ denotes the *l*th covariate with time-varying patterns and nonlinear effects; *L* is the total number of covariates that present nonlinear features;
— $X_{irt}$ and $X_{ist}$ are the pair of interaction covariates in county *i* on day *t*, including the temporal and policy-related interaction among different states; *R* and *S* are the numbers of variables with interactive effects; ${\tilde{\;f}}_{ir}(.)\times {\tilde{\;f}}_{is}(.)$ are the interaction smooth functions with penalties on each null space component;
— *b_i_* is the random effect vector of a state and assumed to follow a Gaussian distribution, noted as $N(0,\sigma^{2})$; $\overset{\prime }{\;f_{i}}(.)$ is the spline function penalized by a ridge penalty that varies across different states; $\vartheta_{it}$ is the error term in state *i* on day *t*.

The control variables include the COVID-19-related features (daily number of newly confirmed coronavirus cases in the state and that number in the adjacent states), policy-related features (whether the order has been issued and the levels of enforcements) and other factors contributing to the variation (i.e. random effect of time and states, weekday–weekend variation and state governor approval rates). The additive terms, such as the random effects, interaction effects and nonlinear time-varying patterns, are also added as variables in the GAM. A detailed summary of the dependent and control variables is reported in electronic supplementary material, Appendix, Section II.

## 5. Results

Our model predicts the daily average number of trips and daily average PMT across all states with relatively high accuracy (*R*^2^(adj.) = 0.882 and 0.919, respectively). Table 1 reports the estimation results of the two models.

**Table 1.** The estimation results of the GAM models. bs, function used in the definition of smooth terms; e.d.f., estimated degrees of freedom; *F*, *F*-test value; fs, factor smooth interactions; re, random effects; s, smooth term.

models	daily average number of trips per person		daily average person-miles travelled	
parametric coefficients	estimate (s.e.)	*t*-value	estimate (s.e.)	*t*-value
(intercept)	0.025 (0.023)	1.051	−2.690 (0.488)	−5.511***
stay-at-home order issued without penalty or without specifying enforcement	−0.122 (0.026)	−4.774***	−1.503 (0.506)	−2.971**
stay-at-home order issued and enforced with warning, and possible fine for repeated offence	−0.125 (0.025)	−4.936***	−0.883 (0.503)	−1.754.
stay-at-home order issued and enforced with fine and possible jail time	−0.167 (0.024)	−6.865***	−1.311 (0.484)	−2.710**
daily number of newly confirmed coronavirus cases in the states (1000)	−0.031 (0.010)	−3.194**	0.304 (0.187)	1.629
daily number of newly confirmed coronavirus cases in the adjacent states (1000)	−0.013 (0.010)	−1.264	0.063 (0.200)	0.316
daily number of newly confirmed coronavirus cases in the USA (1000)	−0.028 (0.002)	−12.140***	−0.282 (0.039)	−7.179***
state governor approval rate (%)	−0.001 (0.001)	−0.402	−0.021 (0.032)	−0.669
weekend	0.140 (0.035)	3.998***	1.823 (0.787)	2.317*
approximate significance of smooth terms	e.d.f.	*F*	e.d.f.	*F*
s (time index)	8.839	566.409***	8.849	490.081***
s (week)	4.064	61.017***	4.708	42.043***
s (state, bs = ’re’)	0.870	0.019***	0.009	0.000***
s (time index, state, bs = ’fs’)	108.031	4.883***	122.700	11.454***
s (stay at home order, state, bs = ’re’)	19.430	0.325***	27.100	0.566***
model goodness-of-fit				
*R*^2^ (adj.)	0.882		0.919	
deviance explained	0.887		0.923	

*p* < 0.1; *p < 0.05; ** *p* < 0.01; ***p < 0.001.

The most interesting and important findings are three-fold.

**Figure 4.**
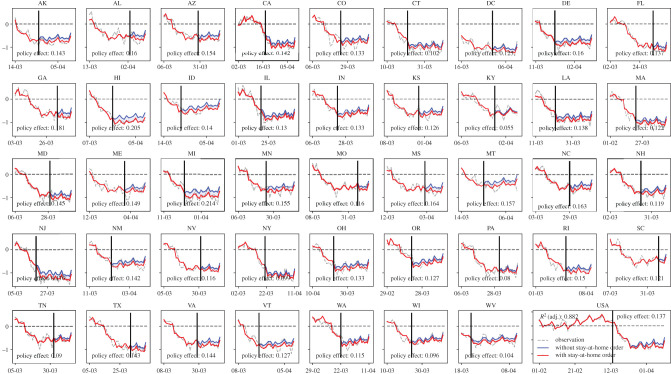
Estimated daily trip increase/reduction per person at the national level and for each state, and the quantified policy effect of stay-at-home orders. In each subfigure, the red curve indicates the model prediction of the daily trip increase/reduction per person assuming with stay-at-home orders; the blue curve indicates the model prediction assuming without stay-at-home orders; policy effect indicates the estimated reduction in the daily number of trips per person that is solely attributed to the effect of stay-at-home orders; the solid vertical line indicates the effective date of the orders.

**Figure 5.**
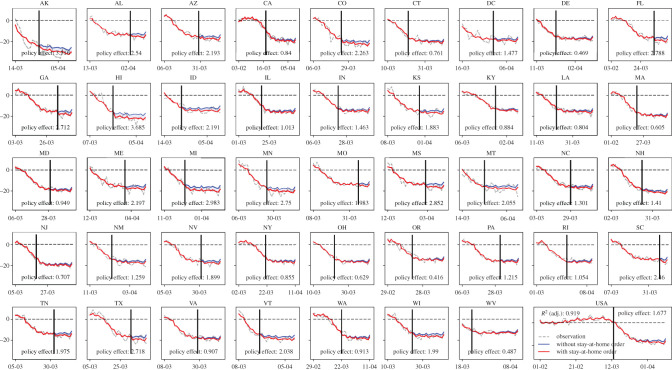
Estimated daily person-miles travelled increase/reduction at the national level and for each state, and the quantified policy effect of stay-at-home orders. In each subfigure, the red curve indicates the model prediction of the daily person-miles travelled increase/reduction assuming with stay-at-home orders; the blue curve indicates the model prediction assuming without stay-at-home orders; policy effect indicates the estimated reduction in the daily number of trips per person that is solely attributed to the effect of stay-at-home orders; the solid vertical line indicates the effective date of the orders.

*A spontaneous mobility change and the ‘floor’ on human mobility* are found in all states and captured by the models. We apply the estimated models to predict state-level human mobility (illustrated by the red curves in figures 4 and 5) during the COVID-19 period and compare it with the observed human mobility data (illustrated by the grey curves in figures 4 and 5). The prediction captures the trend accurately because of the model's specialty in handling the linear and nonlinear effects at the same time. Both the data and the models depict a strong spontaneous reduction in mobility before the government orders (issuance time indicated by the solid vertical line in each subfigure) and limited mobility reduction after the order issuance. For example, the first subfigure of figure 4 indicates that the average number of trips per person in Alaska had already been reduced by 0.8 trips one week before the stay-at-home order went into effect.

*A limited effect of government orders* for the nation and each state is quantified by the models. The models are applied to estimate the trip reduction and miles travelled reduction due to the issuance of stay-at-home orders by comparing the predictions with and without stay-at-home orders via a partial dependence plot (see electronic supplementary material, Appendix, Section II for more details) approach. As depicted in figures 4 and 5, only a limited number of reductions in human mobility can be attributed to the effect of stay-at-home orders. Still, taking the first subfigure of figure 4 as an example, the blue curve represents the modelled counterfactual effect with the stay-at-home order assumed away; the average gap between the predicted curve (i.e. the red curve) and the counterfactual curve suggests that the policy has led to an average reduction of 0.14 trips in Alaska (i.e. the annotation in the first subfigure). Nationally speaking, the orders lead to a daily reduction of 0.136 trips per person (or 4.9% of the average daily number of trips per person) and a daily reduction of 1.185 PMT (or 4.8% of average daily PMT).

*State heterogeneity in the mobility responses* to government order is quantified. The states that have the most trip reduction and person-mile reduction due to the government orders are ranked in table 2 (a full ranking is offered in electronic supplementary material, Appendix, Section II). The mobility response is greater in states with more strict enforcement, such as Hawaii (the order leads to 0.20 person trips reduced and 3.7 miles reduced), Michigan and Georgia. Compared with states that issued stay-at-home orders without penalty or without specifying particular enforcement, those enforced with a warning and possible fines for repeated offences see an additional 0.4% in trip reduction. Enforcement with fines and possible jail time will further reduce daily trips by 1.5% per person. States such as New Jersey see a more significant reduction in the number of trips while Florida and Alaska have more reduction in PMT quantified, suggesting highly heterogeneous mobility responses to the state policy.

**Table 2.** State ranking in stay-at-home orders' effectiveness.

		trip reduction	miles reduction
*states that have the most trip reduction owing to government orders*			
1	Michigan	0.21	3.0 (rank: 3)
2	Hawaii	0.20	3.7 (rank: 1)
3	Georgia	0.18	2.7 (rank: 8)
4	New Jersey	0.18	0.7 (rank: 38)
5	Mississippi	0.16	2.9 (rank: 4)
*states that have the most mile reduction owing to government orders*			
1	Hawaii	0.20 (rank: 2)	3.7
2	Alaska	0.14 (rank: 17)	3.5
3	Michigan	0.21 (rank: 1)	3.0
4	Mississippi	0.16 (rank: 5)	2.9
5	Florida	0.14 (rank: 22)	2.8

## 6. Discussion

Understanding and accurately predicting human mobility during a pandemic is critical for the control of the spread of COVID-19 and other highly contagious diseases. Here, national, anonymized and privacy-enhanced location-based service data provide empirical evidence on how people in the USA moved during the COVID-19 outbreak and successfully supported human mobility analytics and modelling. The models were deemed statistically significant and accurate, and estimated the effect of state-issued stay-at-home orders and other influential factors on human mobility changes. Dynamics in human mobility, such as distance travelled and the number of trips made in a day, were thus quantified. These model outputs can be immediately integrated into epidemic models that need empirical data support on human movement to assess the transmission of disease and control measures.

The longitudinal models suggest that the states' stay-at-home policies only led to about a 5% reduction in average daily human mobility, revealing its marginal effect in further restricting mobility. The limited effectiveness of the policy can be attributed to two sources: (i) the spontaneous mobility reduction phenomena that occurred before the state-level policy implementation and (ii) a ‘floor’ on human mobility that the average daily number of trips per person has never fallen by more than 23% across the nation. Both the data analytics and the model predictions reinforced these two findings.

It took more than five weeks after the first infection confirmed on *Diamond Princess*, an American cruise ship, for the states to begin implementing stay-at-home orders. The orders were issued too late and accomplished much less than the spontaneous mobility change. A more proactive policy could have generated a stronger effect and flattened the curve further, especially when pharmaceutical interventions were not readily available.

Individuals have reduced the number of trips and trip distance because of stay-at-home orders, but almost two-thirds of the national population are still making trips of more than 1 mile away from home daily. This ‘floor’ on human mobility is contributed by commutes of essential businesses and service providers, and trips to access necessities such as healthcare, food and groceries, as well as outdoor activities to keep mentally and physically healthy. Our data analytics suggested that the reduction in the average daily PMT was more significant than the reduction in the daily number of trips, indicating that more short-distance trips were made. These could be short residential trips, outdoor activities or buying groceries and fast food, as confirmed in other parallel analyses [18,33]. Government agencies could further improve the effectiveness of the stay-at-home orders by educating the general public and increasing enforcement. On the other hand, the depicted ‘floor’ on mobility speaks to the value of human mobility, for which people were willing to venture out. In this regard, working with employers and communities through educational campaigns about the use of facial masks and practising social distancing, and supporting vulnerable populations who may encounter challenges in meeting stay-at-home requirements, can lead to significant societal benefits.

The limitations of the study are fully acknowledged. First, human mobility is one proxy to measure the risks of disease transmission in a region. The actual risks could vary at different point-of-interest (POI) locations and for different activity types and duration, essentially depending on the extent of people gathering and contacting [34]. Future research could further illustrate those details about human travel and activities based on POIs to assess the risks of contraction. Second, the location data sources employed in this study may not fully capture the mobility of the younger and older generations, as well as the lower income population, as the smartphone technology penetration for these population groups could be lower. To address this issue, additional weighting and validation processes can be explored based on land use and sociodemographic information. Last but not least, this study focused entirely on state-level directives. In a few states, local agencies reacted differently. For example, several counties in Utah and Oklahoma implemented stay-at-home orders while the states did not. The Bay Area imposed the policy earlier than the state of California. These local legislations are omitted from our state-level models, which may lead to slight biases towards the spontaneous mobility reduction found in the analysis. As our immediate next step, the authors will examine the county-level results and models.

## Supplementary Material

Supplementary Materials: Additional Details about the Data, Analytics, and Modeling

## Data accessibility

The data as well as the codes developed and used in the study are shared via a publicly accessible repository on GitHub. The repository link is followed: https://github.com/SonghuaHu-UMD/Mobility_RSIF.
