# Supplementary material for: Mobile device location data reveal human mobility response to state-level stay-at-home orders during the COVID-19 pandemic in the USA
Source: J R Soc Interface. 2020 Dec 16;17(173):20200344. doi: 10.1098/rsif.2020.0344 (PMC7811592; doi:10.1098/rsif.2020.0344)
Supplement: Supplementary Materials: Additional Details about the Data, Analytics, and Modeling [file rsif20200344supp1.docx]

SUPPLEMENTARY MATERIALS:

Additional Details about the Data, Analytics, and Modeling

**SECTION I: Data, Analytics, and Descriptive Statistics**

The authors have employed several mobile device location data sources that collectively cover over 150 million samples in the U.S. on a monthly basis. We first integrated and cleaned the mobile device location data. We then clustered the location points into activity locations and identified home and work locations at the census block group (CBG) level to protect privacy. We examined both temporal and spatial features for the entire activity location list to identify home CBGs and work CBGs for workers with a fixed work location. Next, we applied previously developed and validated algorithms to identify all trips from the cleaned data panel, including trip origin, destination, departure time, and arrival time. If an anonymized individual in the sample did not make any trip longer than one mile in distance, this anonymized individual was considered as staying at home. A multi-level weighting procedure expanded the sample to the entire population, using device-level and trip-level weights, so the results are representative of the entire population in a nation, state, or county. The data sources and computational algorithms have been validated based on a variety of independent datasets such as the National Household Travel Survey and American Community Survey, and peer reviewed by an external expert panel in a U.S. Department of Transportation Federal Highway Administration’s Exploratory Advanced Research Program project, titled “Data analytics and modeling methods for tracking and predicting origin-destination travel trends based on mobile device data”. Mobility metrics were then integrated with COVID-19 case data, and population data. Fig. S1 shows a summary of the methodology.

Figure S1. Methodology

## The methodology includes the following spatial and temporal data analytics steps that are critical in identifying human mobility successfully:

***State-of-the-practice data processing***: Some common issues, such as unordered and duplicated records, need careful treatment before extracting any information from mobile device location data. The state-of-the-practice methods for raw data cleaning and quality control often include identifying and merging duplicate device observations, removing outliers, and checking on the obvious data consistency issues (e.g., devices with unreasonably high-speed readings). Figure S2 shows a general data cleaning procedure for mobile device location data taken by the research team based on the four dimensions of data quality assessment: consistency, accuracy, completeness, and timeliness (Batini et al., 2009).


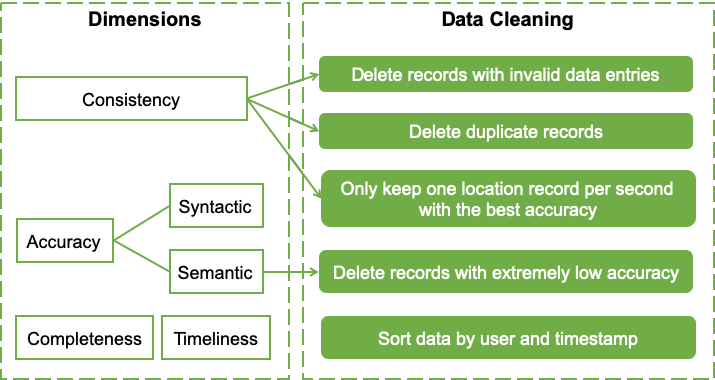


Figure S2. State-of-the-practice data cleaning procedure

The completeness dimension cannot be considered without prior knowledge of the actual individual movements and mobile device usage. The timeliness is addressed by using daily feeds of mobile device location data for our application. For the first two dimensions related to data cleaning, the consistency dimension defines certain semantic rules that a set of data items should obey. A common type of semantic rule is integrity constraints. For example, the latitude and longitude of a location observation should be within a reasonable range. According to the integrity constraints, the cleaning procedure first deletes records with invalid entries and duplicate records to reduce redundancy. Since one subject cannot be at more than one place at the same time, the procedure keeps only one location record per second (with the highest accuracy, if applicable). Another important dimension of data quality assessment is accuracy, including syntactic and semantic accuracy. The syntactic accuracy measures the closeness of a value to all the elements of its corresponding definition domain. The semantic accuracy measures the closeness of a value to its real-world value. For example, an accuracy of 10 meters in a location sighting indicates that the subject should be within a radius of 10 meters from the observed location with a certain confidence level, e.g., 95%. Therefore, the cleaning procedure removes the noisy records with extremely poor accuracy, e.g., two miles.

Location data providers describe their sample sizes with statistics such as daily active users (DAU) and monthly active users (MAU). MAUs are devices that are observed at least once a month and DAUs are devices that are continuously observed throughout the month. Reported data coverage by major data providers ranges between 5% to 70%, depending on whether they report MAU or DAU and how they define active users. While the overall sample size is measured by daily and monthly active users, these measures do not take into consideration that some devices may provide many sightings every day while other devices may only provide a few sightings in a very small number of days. **Table S1** presents more information about the mobile device location dataset used in this research. The following definitions describes the variables presented in the table:

- Population coverage: number of devices with identifiable home census block group (CBG) divided by the population of the study area.
- Temporal consistency: average number of days a device is observed in the study period.
- Frequency: the average location observations per device per day.
- Geographical representativeness: variance of population coverage among different zones of the study area, measured by a Gini coefficient between 0 and 1, with 0 indicating equal sampling rate in all zones and 1 indicating that all observed devices are from a single zone.
- Device representativeness: a measure of the variance in the location point frequency among observed devices. This measure shows if observed devices are comparable in terms of their data frequency and are also measured by a Gini coefficient falling between 0 and 1. Raw data representativeness has a lower value if all observed devices have more consistent data frequency.
- Hourly and daily temporal coverage: a measure of the variation of the number of location point observations among different hours of the day and different days of the month, respectively. Lower values between 0 and 1 indicate a more equitable distribution.

Table S1. Data quality comparison among three commercial LBS datasets

| **Selected Raw Data Quality Metrics** | **Mobile Device Location Dataset** |
| --- | --- |
| **Population coverage (%)** | 23.92 |
| **Geographical representativeness (0~1)** | 0.09 |
| **Frequency (observations per device per day)** | 190 |
| **Temporal consistency (days per device)** | 14.67 |
| **Device representativeness (0~1)** | 0.67 |
| **Hourly temporal coverage (0~1)** | 0.249 |
| **Daily temporal coverage (0~1)** | 0.03 |

***Trip identification***: Trips are not initially included in any mobile device location data sources. Instead, location sightings are continuously generated while the sample device moves, stops, stays static, or starts a new trip. As a result, we developed a trip identification algorithm, which can detect which location sightings form a trip together. We first sort device observations by time. The algorithm assigns a random ID to each trip it identifies. Many location points in the dataset may belong to no trips. The algorithm assigns “0” to the trip ID of these locations to tag them as static points. For every location point, we calculate distance, time, and speed between the point and its immediate previous and next points, if exist. Three hyperparameters need to be set for the algorithm: distance threshold, time threshold, and speed threshold. The speed threshold us used no identify if a location point is recorded on the move. The distance and time threshold are used to identify stay locations and trip ends. At this step, the algorithm identifies the device’s first observation with *speed from≥speed threshold*. This identified location point is recorded on the move, so a hashed trip ID is generated and assigned to this point. All points recorded before this point, if exist, are set to have “0” as their trip ID. Next, a recursive algorithm identifies if the next points are on the same trip and should have the same trip ID.

Then, a recursive algorithm has been developed to check every point to identify if they belong to the same trip as their previous point. If they do, they are assigned the same trip ID. If they do not, they are either assigned a new hashed trip id (when their *speed from≥speed threshold*) or their trip ID is set to “0” (when their *speed from<speed threshold*). Identifying if a point belongs to the same trip as its previous point is based on the point’s “*speed to*”, “*distance to*” and “*time to*” attributes. If a device is seen in a point with *distance to≥distance threshold* but is not observed to move there (*speed to<speed threshold*), the point does not belong to the same trip as its previous point. When the device is on the move at a point (*speed to≥speed threshold*), the point belongs to the same trip as its previous point; but when the device stops, the algorithm checks the radius and dwell time to identify if the previous trip has ended. If the device stays at the stop (points should be closer than the distance threshold) for a period of time shorter than the time threshold, the points still belong to the previous trip. When the dwell time reaches above the time threshold, the trip ends, and the next points no longer belong to the same trip. The algorithm does this by updating “time from” to be measured from the first observation in the stop, not the point’s previous point. The algorithm may identify a local movement as a trip if the device moves within a stay location. To filter out such trips, all trips that are shorter than 300 meters are removed.

## *Activity Clustering:* The algorithm first applies HDBSCAN clustering algorithm to clusters all device observations into activity locations. This step takes the cleaned multi-day location data as input and applies an iterative algorithm until no cluster has a radius larger than two miles. The iterative algorithm consists of two parts: HDBSCAN based on a minimum number of point parameters and filtering non-static clusters based on time and speed checks. After finalizing the potential stay clusters, the algorithm combines nearby clusters to avoid splitting a single activity.

***Home and Work Identification:*** A typical methodology for identifying home and work clusters is to identify the most frequently visited clusters during the night and during the day. Here, instead of setting a fixed time period for each type, e.g., 8pm to 8am as the study period for home CBG identification and the other half day for work CBG identification, the framework examines both temporal and spatial features for the entire activity location list. The benefits are two-fold: the results for workers with flexible or opposite work schedules would be more accurate and the employment type for each device could be detected simultaneously.

***Weighting*:** The sample data needs to be expanded to produce population-level statistics. The devices available in our dataset represent a sample of the population, so device-level weights are needed to expand the device sample. Also, for an observed device, only a sample of all trips may be recorded, so trip-level weights are needed as well. For the sake of timeliness, we have applied simple weighting methods to obtain county-level device weights and state-level trip weights. In order to obtain device-level weights, we have used the home county, obtained from the imputed home CBG information. The weight for each device is equal to the number of devices observed in the device’s imputed home county divided by the population of the county, so all devices residing in a county would have the same device-level weight. For instance, if our sample includes 100 devices in a county with a population of 2,000, each device would be assigned a weight of 20. For trip-level weights, we have calculated number of trips per person (trip rate) for each state during an average weekday in the first two weeks of February from our sample. We have also calculated this trip rate number for each state from the 2017 National Household Travel Survey. We have used a single trip rate for all trips generated from each state, equal to the NHTS trip rate divided by our observed trip rate.

We will share the state-level data analyzed in this paper with the audience. Table S2 shows the relative change from the benchmark (i.e., January 2020) in Average Number of Trips per Person and Average Person-Miles Traveled (PMT) per state, three, two and one week before and one week after each “Stay-at-home” order took effect.

- Starting from as early as two weeks before each state officially issued the “Stay-at-home” order, 21 over 43 states experienced more than 10% average PMT drop compared to the January benchmark. And an average of 15% decrease in average PMT (9% decrease in number of trips per person) across all 43 states compared to previous week are observed. These indicate that people were actively taking actions by reducing their travel prior to the issuance of the orders from the state governments.
- Observation on one week before the “Stay-at-home” orders further shows more decrease in PMT, where all 43 states were observed more than 10% PMT drop, and an average of 17% decrease in average PMT (10% decrease in number of trips per person) across all 43 states compared to previous week are observed.
- One week after each state issued its own “Stay-at-home” order, all 43 states experienced more than 30% average PMT drop, and an average of additional 11% decrease in average PMT (6% decrease in number of trips per person) across all 43 states compared to previous week are observed.

Another finding is that the amount decrease in average PMT is larger than that of average number of trips per person, with an average of 6.5%, 13.8% and 18.6% more decrease across all 43 states for two weeks before, one week before and one week after the “Stay-at-Home” orders are issued. One possible reason is that even though people reduce long-distance or commute trips, they tend to do more short-distance activities such as walking their dogs, jogging and other outdoor exercise activities. As most states’ “Stay-at-Home” allow outdoor exercises, the results indicate that people do follow the orders to some extent.

**Table S2**: **Relative change from the human mobility of January 2020 in Average Number of Trips per Person (# of Trips) and Average Person-Miles Traveled (PMT) per state. Stats for three, two and one week before and one week after each “Stay-at-home” order took effect are shown.**

| **State** | **Three weeks before** | | **Two weeks before** | | **One week before** | | **One week after** | |
| --- | --- | --- | --- | --- | --- | --- | --- | --- |
|  | # of Trips | PMT | # of Trips | PMT | # of Trips | PMT | # of Trips | PMT |
| AL | -1.5% | -7.1% | -12.1% | -24.3% | -12.2% | -27.4% | -16.3% | -32.7% |
| AK | 4.3% | 0.2% | -2.0% | -14.7% | -17.1% | -40.8% | -21.3% | -51.4% |
| AZ | 3.6% | 3.2% | -9.1% | -17.6% | -13.2% | -30.5% | -16.3% | -36.3% |
| CA | 6.4% | 5.1% | 3.8% | 1.9% | -5.6% | -12.0% | -21.3% | -34.0% |
| CO | 7.2% | 3.3% | -1.6% | -8.2% | -17.5% | -33.3% | -21.4% | -44.6% |
| CT | 5.7% | 6.6% | 1.7% | -4.4% | -16.7% | -31.6% | -27.9% | -45.5% |
| DE | 6.2% | 5.8% | 1.2% | -5.4% | -14.8% | -28.9% | -23.2% | -40.4% |
| DC | -8.3% | -8.1% | -25.6% | -38.0% | -32.3% | -55.5% | -33.0% | -58.9% |
| FL | -4.8% | -6.8% | -19.3% | -28.6% | -23.3% | -38.1% | -26.3% | -43.3% |
| GA | -1.7% | -7.1% | -14.8% | -26.0% | -15.2% | -31.0% | -19.4% | -37.8% |
| HI | 4.1% | 0.5% | -0.6% | 1.5% | -14.6% | -27.4% | -26.2% | -51.5% |
| ID | 7.2% | 10.0% | 3.5% | -0.6% | -5.1% | -15.1% | -14.1% | -30.6% |
| IL | 8.7% | 10.3% | 7.7% | 8.1% | -8.3% | -12.1% | -24.4% | -34.8% |
| IN | 7.9% | 9.7% | 1.9% | 1.5% | -11.9% | -20.3% | -17.9% | -36.0% |
| KS | 3.4% | 9.5% | -11.2% | -17.8% | -14.9% | -33.5% | -18.3% | -38.9% |
| KY | 6.7% | 7.5% | -3.7% | -5.1% | -15.4% | -24.9% | -14.2% | -31.2% |
| LA | 6.4% | 7.8% | 4.9% | 2.4% | -11.0% | -19.2% | -19.5% | -31.3% |
| ME | -1.7% | -1.7% | -16.3% | -28.4% | -19.2% | -34.2% | -21.3% | -39.6% |
| MD | 1.5% | -3.5% | -14.8% | -28.7% | -25.8% | -44.1% | -26.6% | -48.5% |
| MA | 5.3% | 5.7% | -1.0% | -6.9% | -21.0% | -35.5% | -29.8% | -49.5% |
| MI | 7.5% | 13.3% | 2.4% | 1.2% | -12.7% | -24.5% | -27.1% | -47.0% |
| MN | 7.7% | 14.5% | -3.1% | -5.2% | -16.3% | -32.8% | -20.0% | -44.0% |
| MS | -0.6% | -0.4% | -13.6% | -23.4% | -12.3% | -25.0% | -17.1% | -31.6% |
| MO | -9.6% | -15.0% | -16.0% | -30.6% | -15.2% | -32.5% | -14.3% | -32.1% |
| MT | 6.0% | 6.4% | -1.5% | -12.7% | -6.8% | -24.6% | -15.1% | -36.9% |
| NV | 0.9% | -4.5% | -18.8% | -26.4% | -21.5% | -34.4% | -22.5% | -39.8% |
| NH | 5.1% | 6.1% | -6.1% | -12.7% | -18.6% | -36.8% | -23.8% | -45.2% |
| NJ | 5.1% | 5.7% | 4.2% | -0.1% | -14.0% | -24.3% | -31.3% | -47.6% |
| NM | 7.4% | 8.0% | 4.7% | 0.5% | -9.1% | -19.5% | -15.7% | -33.1% |
| NY | 5.3% | 5.1% | 2.5% | -0.5% | -15.7% | -23.5% | -32.3% | -45.2% |
| NC | 4.8% | 1.4% | -8.0% | -20.2% | -13.0% | -31.6% | -15.2% | -36.5% |
| OH | 6.7% | 7.3% | 2.7% | 0.0% | -13.5% | -23.3% | -21.3% | -39.4% |
| OR | 6.7% | 7.6% | 3.6% | -2.5% | -4.2% | -14.3% | -17.9% | -33.7% |
| PA | -0.5% | -3.0% | -21.0% | -30.7% | -26.5% | -42.1% | -23.8% | -42.9% |
| RI | 3.9% | 1.7% | -11.2% | -19.3% | -21.0% | -36.7% | -25.9% | -44.2% |
| SC | -9.8% | -20.2% | -11.8% | -27.9% | -10.9% | -32.1% | -11.8% | -34.4% |
| TN | -1.7% | -0.4% | -14.6% | -22.4% | -13.3% | -28.9% | -13.8% | -33.3% |
| TX | -2.6% | 0.2% | -17.0% | -25.4% | -21.4% | -34.1% | -24.1% | -37.7% |
| VT | 3.8% | 14.3% | -1.6% | 4.3% | -16.7% | -30.8% | -25.5% | -43.8% |
| VA | 2.6% | -1.1% | -12.1% | -25.6% | -19.3% | -38.5% | -20.6% | -42.5% |
| WA | 5.9% | 6.9% | 1.2% | -2.2% | -8.7% | -19.5% | -21.6% | -38.3% |
| WV | 6.4% | 6.4% | 2.8% | 0.5% | -11.4% | -20.5% | -16.6% | -31.2% |
| WI | 7.6% | 12.7% | 2.2% | 3.2% | -16.7% | -23.6% | -20.6% | -38.1% |

**SECTION II: Materials and Methods**

***2.1. Model Description***

This section provides a detailed description of the GAM we employed to examine the policy effects on human mobility change. GAM is a semi-parametric model with a linear predictor involving a series of additive non-parametric smooth functions of covariates. Compared to the classical ordinary least squares (OLS) regression, GAM is more flexible with fewer assumptions, which is useful when data cannot meet OLS assumptions, such as independence, normality, and homogeneity. Additionally, a noticeable advantage of GAM lies in its capability and flexibility to handle different formats of nonlinear effects (Wood, 2003). By changing the spline functions, various effects can be captured under one model framework, including the random effects, the interaction relationships, and the spatiotemporal autocorrelations.

As a longitudinal analysis with repeated observations over time for each state, the non-independence among the repeated observations and the heterogeneous variability over time should be carefully addressed. Mixed (also named multilevel) models are widely used to handle the panel data (Wolfinger and O'connell, 1993). However, traditional mixed models are linear-based and fail to obtain high performance under data with significant nonlinear fluctuation. Hence, a GAM structure is involved to handle the panel data, with several additive smooth terms besides the linear fixed effect to address the heterogeneous covariance structures. To specific, the additive terms including:

1. Random effects across all states, to capture the unobserved heterogeneity.
2. Interactions between stay-at-home order and state, to capture the varying effect of policies across different states;
3. Time-varying patterns, including an average changing pattern and a seasonally changing pattern (weekly patterns), to fit the autoregressive time series;
4. Spatiotemporal interactions, to capture the spatiotemporal heterogeneity over time across different states.

GAM is estimated using the R ‘mgcv’ package (Wood, 2017). Variance components are estimated by the Restricted Maximum Likelihood Estimation (REML), which is widely used in models with random effects. The expression of GAM is shown as follows:

|  | $T_{i}=\beta_{0}+\sum_{k=1}^{K} \beta_{ik}X_{ik}+\sum_{l=1}^{L} f_{il}\left( X_{il} \right)+\sum_{r=1}^{R} \sum_{s=1}^{S} \tilde{f}_{ir}\left( X_{ir} \right)\times\tilde{f}_{is}\left( X_{is} \right)+\acute{f_{i}}\left( b_{i} \right)+\vartheta_{i}$ | S (3.1) |
| --- | --- | --- |

where $T_{i}$ is the vector of the average number of trips per person or average person-miles traveled in state *i* over different days; $\beta_{0}$ is the overall intercept; $\beta_{ik}$ is the *k^th^* coefficient of fixed effects that vary across different states; *K* is the total number of fixed effects; $X_{ik}$ refers to the *k^th^* fixed covariate; *L* is the total number of covariates that present nonlinear features; $f_{i}(.)$ is a low rank isotropic smooth function and $X_{il}$ denotes the *l^th^* covariate with nonlinear effects; the time-varying patterns are included in$X_{il}; X_{ir}$ and $X_{is}$ are the *r^th^* pair of interaction covariate, including the temporal and policy-related interaction among different states; *R* and *S* are the numbers of variables with interactive effects; $\tilde{f_{i}}(.)$ is an interaction smooth functions with penalties on each null space component; $b_{i}$ is the random effect vector of a state, and assumed to follow a Gaussian distribution, noted as $N(0,\sigma^{2})$; $\acute{f_{i}}\left( . \right)$ is the spline function penalized by a ridge penalty varies across different states; $\vartheta_{i}$ is the error term in state *i*.

***2.2. Variable Description***

Two dependent variables are considered, i.e. the Daily Average Number of Trips Per Person (TPP) and the Daily Average Person-Miles Traveled (PMT), which are used to represent the changes of state-level individual travel frequency. All the dependent variables are the relative value using the corresponding values in January as reference. In other words, they are the increase compared with the same day of the week in January:

|  | $\check{Y}_{d}=Y_{d}-\bar{YR}_{k\vert W_{k}=W_{d}}$ | S (3.2) |
| --- | --- | --- |

where $\check{Y}_{d}$ is the relative dependent variables in day *d*; $Y_{d}$ is the absolute dependent variables in day *d;*$W_{d}$ is the week of day *d*; $\bar{YR}_{k|W_{k}=W_{d}}$ is the average value of TPP or PMT in days belonging to the week $W_{d}$in January.

Independent variables include the policy-related features, such as the stay-at-home order with different level of enforcement and the state government approval rate; the cases-related features, such as the daily new cases in the state, the adjacent states, and the nationwide cases; and the temporal variables, such as the time index, the week, and whether it is weekend.

The variance inflation factor (VIF) is used to check for multicollinearity, and variables with VIF values greater than 5.0 were excluded. It is worth mentioning highly multicollinearity is observed between the number of new cases and the accumulated cases, and thus the accumulated number of cases is excluded. Similar high multicollinearity is observed between stay-at-home order and COVID-19 Emergency Declaration, and we keep the stay-at-home order in the final models.

The summary of variables is reported in Table S3. The average of TPP and PMT are both negative, indicating the trip frequency and trip miles are both presenting the decreasing trends. The large St.D., on the other hand, implying the changes are heterogeneous across different states.

***2.3. Modeling Results and Discussions***

The results of the two GAMs are shown in Table S4 and S5, respectively. Two components are included: the parametric coefficients, corresponding to the linear fixed effects; and the nonparametric smooth terms, corresponding to the nonlinear effects, random effects (bs=’re’), and interaction effects (bs=’fs’). Model fit indexes are 0.882 and 0.919 for the two models, indicating that GAMs fit the data well.

*Linear effects* - the stay-at-home orders present significant negative effects on both the number of trips and the person-miles traveled. With the enforcement becoming more strict, the effects of stay-at-home orders on reducing mobility also increase. For case-related variables, the number of nationwide cases is significantly and negatively correlated with both the number of trips and the person-miles traveled. The number of cases in the states, however, only presents a significantly influence on the number of trips, not on the person-miles traveled. For temporal features, the weekend presents significant positive relationships in two models, indicating the reduction of trips on weekends is less than weekdays (i.e. the increment is greater). We also build in a control variable for the effect of governor approval rate and that is deemed insignificant by the model.

*Nonlinear effects* – the estimated degrees of freedom (e.d.f.) are all largely greater than 1.0, suggesting that strong nonlinearities exist. In addition, the interaction terms in all models present P-values smaller than 0.1, implying these nonlinear effects are statistically significant. The fitted results by the spline functions are shown in Figure S2. The values of the vertical axis show the additive effect of the independent variables on the number of trips and the person-miles traveled.

**Table S3: Summary of Variables in the Models**

| Variable | Description | Mean | St.D. | Min. | 50% | Max. |
| --- | --- | --- | --- | --- | --- | --- |
| Dependent Variables | | | | | | |
| Avg. Number of Trips | Daily Average Number of Trips Per Person | -0.171 | 0.439 | -1.487 | -0.004 | 0.835 |
| Avg. PMT | Daily Average Person-Miles Traveled | -4.367 | 8.900 | -36.003 | -0.526 | 21.072 |
| Independent Variables | | | | | | |
| Stay-at-home order | Categorical Variables.  0: No Stay-at-home order (**Reference**);  1: Stay-at-home order issued without penalty or without specifying enforcement;  2: Stay-at-home order issued and enforced with warning, and possible fine for repeated offense;  3: Stay-at-home order issued and enforced with fine and possible jail time | - | - | - | - | - |
| *FEMA* | *Dummy Variables: 0: No COVID-19 Emergency Declaration; 1: COVID-19 Emergency Declaration issued* | *0.170* | *0.376* | *0.000* | *0.000* | *1.000* |
| New Cases | Daily number of newly confirmed coronavirus cases in the states (1,000) | 0.143 | 0.709 | 0.000 | 0.000 | 11.186 |
| *Sum Cases* | *Daily number of accumulated confirmed coronavirus cases in the states (1,000)* | *1.386* | *8.428* | *0.000* | *0.002* | *181.029* |
| Adj. New Cases | Daily number of newly confirmed coronavirus cases in adjacent states (1,000) | 0.056 | 0.728 | 0.000 | 0.000 | 13.082 |
| *Adj. Sum Cases* | *Daily number of accumulated confirmed coronavirus cases in adjacent states (1,000)* | *0.579* | *8.719* | *0.000* | *0.000* | *205.381* |
| National New Cases | Daily number of newly confirmed coronavirus cases in the nation (1,000) | 7.512 | 11.721 | 0.000 | 0.068 | 35.114 |
| Week | The day of week, from 0 (Monday) to 6 (Sunday) | 3.030 | 2.000 | 0.000 | 3.000 | 6.000 |
| Is Weekend | If the day is weekend, 1; else 0 | 0.296 | 0.457 | 0.000 | 0.000 | 1.000 |
| Time Index | The day difference from the current timestamp to 02/01/2020 | 35.271 | 20.564 | 0.000 | 35.000 | 70.000 |
| Approval Rate | State governor approval rate | 3.359 | 12.390 | 0.340 | 0.520 | 59.000 |

*Italic texts: excluded variables due to multicollinearity.*

**Table S4: Estimated GAM Model of Daily Average Number of Trips Per Person**

| Parametric coefficients: | | | | | |
| --- | --- | --- | --- | --- | --- |
|  | **Estimate** | **Std. Error** | **t value** | **Pr(>\|t\|)** |  |
| (Intercept) | 0.025 | 0.023 | 1.051 | 0.293 |  |
| Stay-at-home order issued without penalty or without specifying enforcement | -0.122 | 0.026 | -4.774 | 0.000 | *** |
| Stay-at-home order issued and enforced with warning, and possible fine for repeated offense | -0.125 | 0.025 | -4.936 | 0.000 | *** |
| Stay-at-home order issued and enforced with fine and possible jail time | -0.167 | 0.024 | -6.865 | 0.000 | *** |
| Daily number of newly confirmed coronavirus cases in the states (1,000) | -0.031 | 0.010 | -3.194 | 0.001 | ** |
| Daily number of newly confirmed coronavirus cases in the adjacent states (1,000) | -0.013 | 0.010 | -1.264 | 0.207 |  |
| Daily number of newly confirmed coronavirus cases in the U.S. (1,000) | -0.028 | 0.002 | -12.140 | 0.000 | *** |
| State governor approval rate | -0.001 | 0.001 | -0.402 | 0.688 |  |
| Weekend | 0.140 | 0.035 | 3.998 | 0.000 | *** |
| Approximate significance of smooth terms: | | | | | |
|  | **e.d.f** | **Ref.df** | **F** | **P-value** |  |
| s (Time Index) | 8.839 | 9.000 | 566.409 | 0.000 | *** |
| s (Week) | 4.064 | 5.000 | 61.017 | 0.000 | *** |
| s (State, bs=’re’) | 0.870 | 48.000 | 0.019 | 0.000 | *** |
| s (Time Index, State, bs=’fs’) | 108.031 | 498.000 | 4.883 | 0.000 | *** |
| s (Stay at home order, State, bs=’re’) | 19.430 | 88.000 | 0.325 | 0.000 | *** |
| Model fit: | | | | | |
| R-sq.(adj) | 0.882 | | | | |
| Deviance explained | 0.887 | | | | |
| -REML | -1418.100 | | | | |
| Scale est. | 0.023 | | | | |

*Note: ‘.’ p<0.1; ‘*’ p<0.05; ‘**’ p<0.01; ‘***’ p<0.001*

The left figures present the time-dependent random effect (with the dash lines showing the confidence interval), which can be deemed as the impact from other unobserved time-varying factors represented as follows:

- A slight mobility drop is captured by the random effect of both models near 2020/02/15, corresponding to the Presidents’ Day weekend.
- A mobility increase is then captured, with a tipping point near 2020/03/07, four days before WHO defined the COVID-19 as a pandemic. In line with others’ data findings (e.g. PlaceIQ 2020), we argue this is due to a model-unobserved panic such that people were stocking up goods for the possible lock-down.
- A sharp decrease occurs between 2020/03/07 and 2020/03/22, followed by a dramatic rebound. The rebounding effect for the daily average person-miles traveled is not as steep. One explanation is that the increased trips mainly belong to short-distance trips, such as the exercises near home locations. This finding is also in line with other most-recent studies (PlaceIQ, 2020; Google, 2020).

The right subplots in Figure S2(a) and S2(b) show the time-varying heterogeneity across different states. With an interaction spline function, these heterogeneities are well captured by the models. Despite all the negative and significant effects from the model variables, states such as DC, NJ, MA, FL, and TX, present additional decreasing trends in heterogeneity, indicating extra caution in these state residents. States such as ID, MT, WY, UT, mostly present increasing trends.


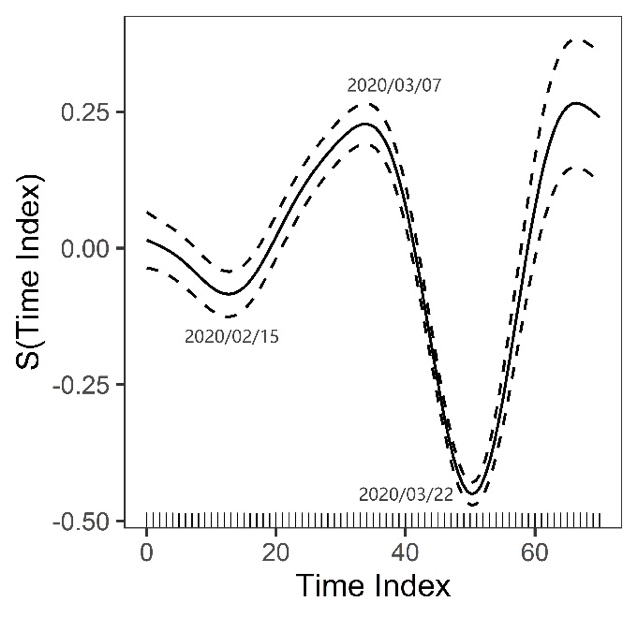

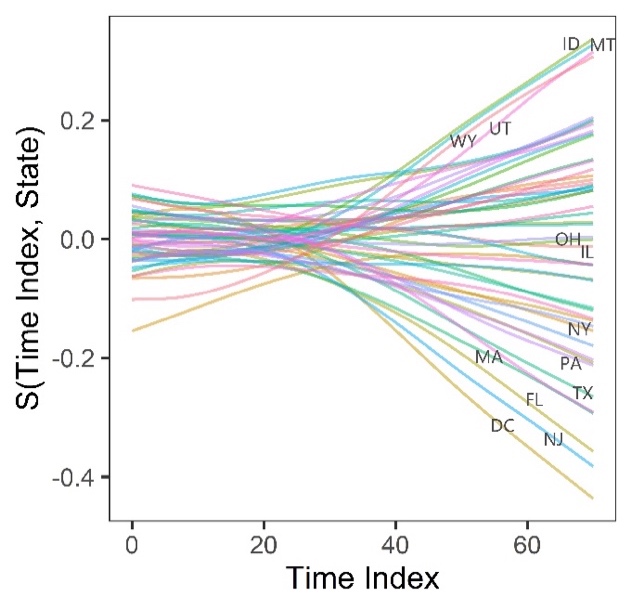


(a) Daily Average Number of Trips Per Person Model (Temporal Effect and State Heterogeneity)


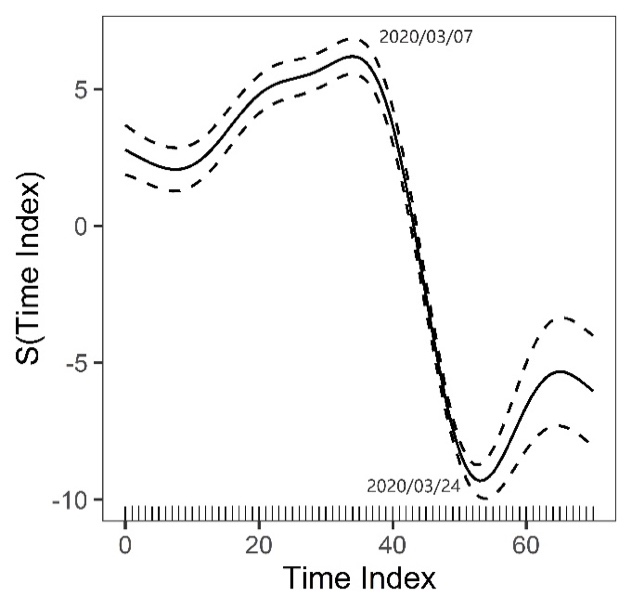

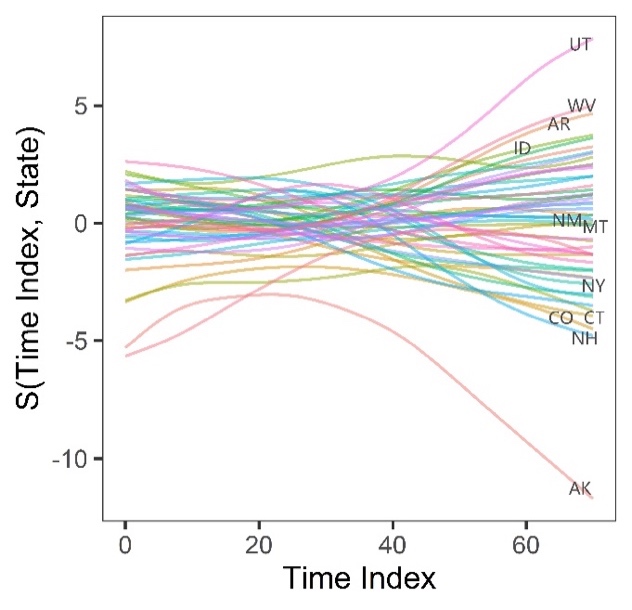


(b) Daily Average Person-Miles Traveled Model (Temporal Effect and State Heterogeneity)

**Figure S2. Nonlinear temporal effects in Daily Average Number of Trips per Person Model (a) and Daily Average Person-Miles Traveled Model (b).**

**Table S5: Estimated GAM Model of Daily Average Person-Miles Traveled**

| Parametric coefficients: | | | | | |
| --- | --- | --- | --- | --- | --- |
|  | **Estimate** | **Std. Error** | **t value** | **Pr(>\|t\|)** |  |
| (Intercept) | -2.690 | 0.488 | -5.511 | 0.000 | *** |
| Stay-at-home order issued without penalty or without specifying enforcement | -1.503 | 0.506 | -2.971 | 0.003 | ** |
| Stay-at-home order issued and enforced with warning, and possible fine for repeated offense | -0.883 | 0.503 | -1.754 | 0.080 | . |
| Stay-at-home order issued and enforced with fine and possible jail time | -1.311 | 0.484 | -2.710 | 0.007 | ** |
| Daily number of newly confirmed coronavirus cases in the states (1,000) | 0.304 | 0.187 | 1.629 | 0.457 |  |
| Daily number of newly confirmed coronavirus cases in the adjacent states (1,000) | 0.063 | 0.200 | 0.316 | 0.752 |  |
| Daily number of newly confirmed coronavirus cases in the U.S. (1,000) | -0.282 | 0.039 | -7.179 | 0.000 | *** |
| State governor approval rate | -0.021 | 0.032 | -0.669 | 0.504 |  |
| Weekend | 1.823 | 0.787 | 2.317 | 0.021 | * |
| Approximate significance of smooth terms: | | | | | |
|  | **e.d.f** | **Ref.df** | **F** | **P-value** |  |
| s (Time Index) | 8.849 | 9.000 | 490.081 | 0.000 | *** |
| s (Week) | 4.708 | 5.000 | 42.043 | 0.000 | *** |
| s (State, bs=’re’) | 0.009 | 48.000 | 0.000 | 0.000 | *** |
| s (Time Index, State, bs=’fs’) | 122.700 | 498.000 | 11.454 | 0.000 | *** |
| s (Stay at home order, State, bs=’re’) | 27.100 | 88.000 | 0.566 | 0.000 | *** |
| Model fit: | | | | | |
| R-sq.(adj) | 0.919 | | | | |
| Deviance explained | 0.923 | | | | |
| -REML | 8634.800 | | | | |
| Scale est. | 6.567 | | | | |

*Note: ‘.’ p<0.1; ‘*’ p<0.05; ‘**’ p<0.01; ‘***’ p<0.001*

***2.4 Partial Dependence Plot (PDP)***

The various additive nonlinear effects contribute to a high performance of model fit. The coefficients in the linear part only present the average fixed effects, however, the random effects across different states are eliminated. Thus, a partial dependence plot (PDP) method is introduced to examine the effects of stay-at-home orders on travel patterns across different states.

PDP is widely used to interpret black-box models like various machine learning methods (Friedman, 2001). It shows the dependence between the response variable and the predictor, marginalizing over the values of all other predictors. In this study, the partial dependence of stay-at-home order is calculated (see Table S6), serving as the policy effect for each state. The plots of PDP are presented in Figure 4 and 5 and discussed in the main text.

| \|  \| $P_{i}=Y_{i}-\hat{Y}_{i}$ \| S(3.3) \| \| --- \| --- \| --- \| |  |
| --- | --- | --- | --- | --- |

where $Y_{i}$ is the predicted number of trips or PMT of the state *i*; $\hat{Y}_{i}$ is the predicted number of trips or PMT of state *i* when the value of the stay-at-order variable is set as zero.

**Table S6: The Model Estimated Policy Effect on Daily Average Trips per Person (TPP) and Daily Person-Miles Traveled (PMT)**

| State | Policy effect on TPP | Policy effect on PMT | Rank of TPP | Rank of PMT |
| --- | --- | --- | --- | --- |
| KY | 0.055 | 0.884 | 1 | 11 |
| PA | 0.080 | 1.215 | 2 | 17 |
| TN | 0.090 | 1.975 | 3 | 25 |
| WI | 0.096 | 1.990 | 4 | 27 |
| NY | 0.099 | 0.855 | 5 | 10 |
| CT | 0.102 | 0.761 | 6 | 7 |
| WV | 0.104 | 0.487 | 7 | 3 |
| WA | 0.115 | 0.913 | 8 | 13 |
| MO | 0.116 | 1.983 | 9 | 26 |
| NV | 0.116 | 1.899 | 10 | 24 |
| NH | 0.119 | 1.410 | 11 | 20 |
| SC | 0.121 | 2.460 | 12 | 34 |
| MA | 0.122 | 0.605 | 13 | 4 |
| DC | 0.125 | 1.477 | 14 | 22 |
| KS | 0.126 | 1.883 | 15 | 23 |
| VT | 0.127 | 2.038 | 16 | 28 |
| OR | 0.127 | 0.416 | 17 | 1 |
| IL | 0.130 | 1.013 | 18 | 15 |
| OH | 0.133 | 0.629 | 19 | 5 |
| IN | 0.133 | 1.463 | 20 | 21 |
| CO | 0.133 | 2.263 | 21 | 33 |
| FL | 0.137 | 2.788 | 22 | 39 |
| LA | 0.138 | 0.804 | 23 | 8 |
| ID | 0.140 | 2.191 | 24 | 30 |
| NM | 0.142 | 1.259 | 25 | 18 |
| CA | 0.142 | 0.841 | 26 | 9 |
| AK | 0.143 | 3.516 | 27 | 42 |
| TX | 0.143 | 2.718 | 28 | 37 |
| VA | 0.144 | 0.907 | 29 | 12 |
| MD | 0.145 | 0.949 | 30 | 14 |
| ME | 0.149 | 2.197 | 31 | 32 |
| RI | 0.150 | 1.054 | 32 | 16 |
| AZ | 0.154 | 2.193 | 33 | 31 |
| MN | 0.155 | 2.750 | 34 | 38 |
| MT | 0.157 | 2.055 | 35 | 29 |
| DE | 0.160 | 0.469 | 36 | 2 |
| AL | 0.160 | 2.540 | 37 | 35 |
| NC | 0.163 | 1.301 | 38 | 19 |
| MS | 0.164 | 2.852 | 39 | 40 |
| NJ | 0.176 | 0.707 | 40 | 6 |
| GA | 0.181 | 2.712 | 41 | 36 |
| HI | 0.205 | 3.685 | 42 | 43 |
| MI | 0.214 | 2.983 | 43 | 41 |
